# Identify Alternative Splicing Events Based on Position-Specific Evolutionary Conservation

**DOI:** 10.1371/journal.pone.0002806

**Published:** 2008-07-30

**Authors:** Liang Chen, Sika Zheng

**Affiliations:** 1 Molecular and Computational Biology, Department of Biological Sciences, University of Southern California, Los Angeles, California, United States of America; 2 Department of Microbiology, Immunology, and Molecular Genetics, Howard Hughes Medical Institute, University of California Los Angeles, Los Angeles, California, United States of America; University of California Berkeley, United States of America

## Abstract

The evolution of eukaryotes is accompanied by the increased complexity of alternative splicing which greatly expands genome information. One of the greatest challenges in the post-genome era is a complete revelation of human transcriptome with consideration of alternative splicing. Here, we introduce a comparative genomics approach to systemically identify alternative splicing events based on the differential evolutionary conservation between exons and introns and the high-quality annotation of the ENCODE regions. Specifically, we focus on exons that are included in some transcripts but are completely spliced out for others and we call them conditional exons. First, we characterize distinguishing features among conditional exons, constitutive exons and introns. One of the most important features is the position-specific conservation score. There are dramatic differences in conservation scores between conditional exons and constitutive exons. More importantly, the differences are position-specific. For flanking intronic regions, the differences between conditional exons and constitutive exons are also position-specific. Using the Random Forests algorithm, we can classify conditional exons with high specificities (97% for the identification of conditional exons from intron regions and 95% for the classification of known exons) and fair sensitivities (64% and 32% respectively). We applied the method to the human genome and identified 39,640 introns that actually contain conditional exons and classified 8,813 conditional exons from the current RefSeq exon list. Among those, 31,673 introns containing conditional exons and 5,294 conditional exons classified from known exons cannot be inferred from RefSeq, UCSC or Ensembl annotations. Some of these *de novo* predictions were experimentally verified.

## Introduction

Alternative splicing is one of the most important mechanisms for higher organisms to expand the information content from genome to transcriptome. Bioinformatics analyses based on EST sequences and exon-exon junction microarray studies show that 59%∼74% of human genes are alternatively spliced [1], [2]. Previous studies estimated that cassette exons make up 53%∼61% of alternative splicing events in most species [3], [4]. Although EST and microarray based studies have made much progress in the prediction of alternative splicing events, they are not sufficient to detect all splice variants due to the biased sampling and the bias and noise inherent to EST preparation and microarray technology. Leparc et al. used splice-site sequence Markov models and a Bayesian classifier to identify novel cassette exons from intron sequences [5]. They successfully predicted and experimentally confirmed 26 novel human cassette exons which are involved in intracellular signaling. Sorek et al. assembled 243 alternative and 1,753 constitutive exons that are conserved between human and mouse [6]. They identified several features differentiating between alternatively spliced and constitutively spliced exons. The most important features are the ones based on the sequence similarity between human and mouse. Yeo et al. used sequence features to distinguish alternative splicing events conserved in human and mouse [7]. Therefore, sequence content and sequence conservation provide alternative ways to study alternative splicing [8].

It has been shown that the evolution rate is lower for exon regions near the intron-exon boundaries than the middle part of exons, by estimating the non-synonymous substitution rate and the synonymous substitution rate from the alignment of human-mouse sequences [9]. The SNP density is the lowest near the splice sites, which also indicates that exon regions near the splice sites are under higher selection pressure [10]. Here, we consider the conservation score of every site of conditional exons, constitutive exons and conditional-exon-free introns. The conservation score is from the PhastCons phylo-hidden Markov model [11] and it is the posterior probability that the site is conserved across 17 vertebrate species. We uncovered the position-specific patterns for the conservation scores and compared conditional exons, constitutive exons and conditional-exon-free introns. The position-specific conservation pattern is more efficient in identifying conditional exons than the overall conservation score of individual exons.

Recently, the pilot project of the Encyclopedia of DNA Elements (ENCODE) [12] has rigorously identified functional elements in the 1% region of the human genome. The GENCODE [13] consortium of the ENCODE project has manually prepared a high-quality annotation for transcripts in the ENCODE regions. In this paper, we utilize the detailed annotation of the ENCODE regions and assemble the lists of conditional exons, constitutive exons and conditional-exon-free introns as training sets. We have two goals: (1) identify novel conditional exons from intron regions; (2) classify known exons into conditional exons and constitutive exons. We used the Random Forests machine learning method [14] to identify novel conditional exons from intron regions and achieved 97% specificity and 64% sensitivity. For classifying exons into conditional exons and constitutive exons, although the sensitivity is only 32%, the specificity can be as high as 95%.

## Results

### Position-specific Conservation Pattern for Exons and Introns

**Figure 1:**
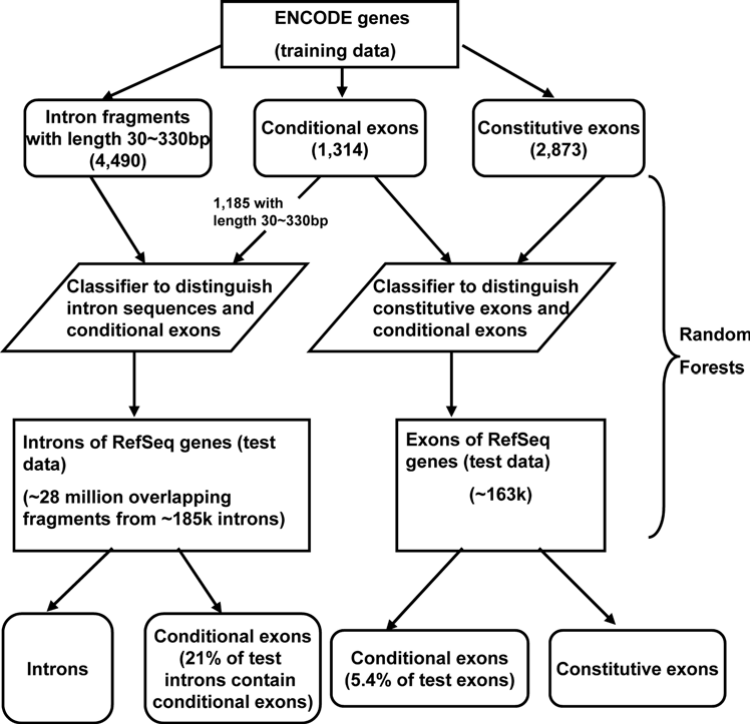
Data flowchart of identifying alternative splicing events based on the ENCODE data and the Random Forests classifiers. Conditional exons are exons that are included in some transcripts but are completely skipped in others. Non-conditional exons are called constitutive exons. Our procedures contain the training with the ENCODE data and the prediction using the Random Forests classifiers.

**Figure 2:**
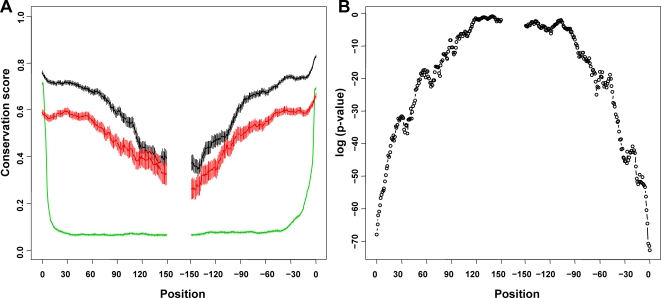
Position-specific conservation scores of exons and introns. (A) Conservation score vs. relative position to splicing site. For every site of exon or intron, x is defined as the position relative to the nearest splice site. It is positive for distances from the 5′ edge and negative for distances from the 3′ edge. Y axis is the average conservation score for constitutive exons (upper lines, black), conditional exons (middle lines, red), and introns (bottom lines, green). The error bar indicates the standard error of the mean for each position. (B) Position-specific p-value for the difference between conditional exons and constitutive exons (log scale). The p-value is based on a one-tailed t test that conditional exons have lower conservation scores than constitutive exons.

In this paper, we are interested in conditional exons that are included in some transcripts but are completely spliced out for other transcripts. These include traditional definition of cassette exons, mutually exclusive exons, retained introns and other complicated alternative splicing events, but not alternative 5′ or 3′ exons. Non-conditional exons are called constitutive exons. Our data flowchart contains the training with the ENCODE data and the prediction using the Random Forests classifiers (Fig. 1). Conditional exons, constitutive exons and conditional-exon-free introns were assembled from the ENCODE regions. Figure 2A plots conservation scores along relative positions of exons or introns. The bias due to the different lengths of exons was corrected in the following way. For each relative position x, the average conservation score was calculated only for exons containing that position. Similar correction was performed for introns. Compared with introns, both conditional exons (red) and constitutive exons (black) have much higher conservations. The conservation scores gradually reduce along the relative positions to exon edges. On the contrary, the conservation scores of introns (green) drop quickly and stayed around 0.07 after about 30 base pair (bp). The 3′ positions of introns (−47 to −7) have relatively higher conservation scores than the 5′ positions of introns (7 to 47) (p-values based on one-tailed t tests≤0.001), probably reflecting the branching point and the poly-pyrimidine tract upstream of the 3′ splice site. Compared with constitutive exons, conditional exons have lower conservation scores and the difference is remarkably more significant for regions near the edges. One-tailed t tests were performed to compare the conservation scores of conditional exons and those of constitutive exons. Figure 2B shows the p-value for each position. It indicates that the selection pressure on the boundaries of conditional exons is significant lower than that for constitutive exons. The difference tends to decrease towards the middle part of exons.

**Figure 3:**
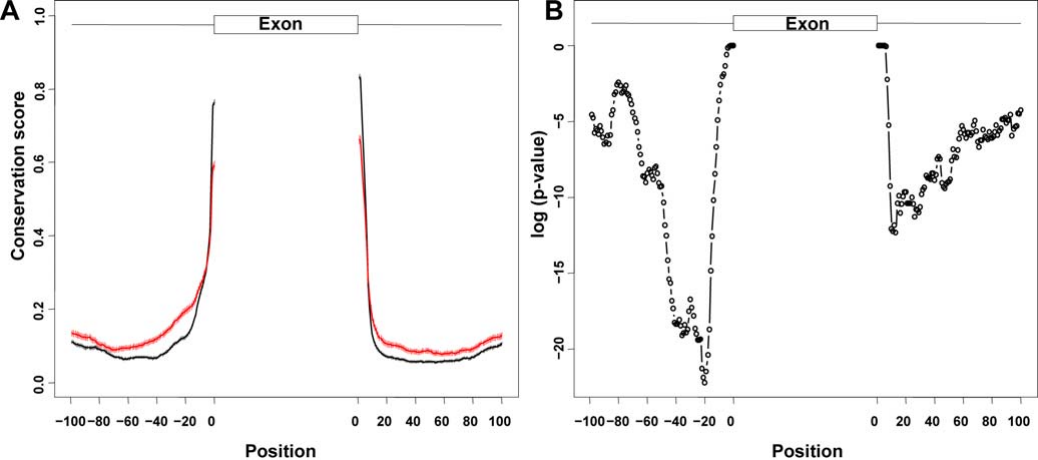
Position-specific conservation scores of flanking intronic regions. (A) Conservation score vs. position. The upper lines (red) are for flanking regions of conditional exons and the bottom lines (black) are for flanking regions of constitutive exons. The error bar indicates the standard error of the sample mean for each position. (B) Position-specific p-value for the difference between conditional exons and constitutive exons (log scale). The p-value is based on a one-tailed t test that the flanking intronic regions of conditional exons have higher conservation scores than those of constitutive exons.

On the contrary, if we consider the conservation scores of flanking intronic regions, intronic regions flanking conditional exons (red) have higher conservation scores than those flanking constitutive exons (black) (see Fig. 3A). Sorek et al. also reported that the intronic regions flanking cassette exons are conserved between human and mouse [6]. In addition, observed from Figure 3A, the differences for upstream intronic regions of exons are larger than those for downstream intronic regions. One-tailed t test was performed to compare the differences for upstream regions (−100, −10) and the differences for downstream regions (10, 100). The p-value is 1.1×10^−6^. Figure 3B plots the position-specific p-values for those differences. The flanking (−46, −18) regions are the most conserved regions for conditional exons (p-values for the differences≤10^−15^). Therefore, the upstream intronic regions and the downstream intronic regions are not symmetric and the upstream intronic regions of conditional exons are much more conserved. All of these results show that there are differences between conditional exons, constitutive exons, and their flanking intronic regions in terms of conservation levels. Moreover, these differences are position-dependent and are functions of the relative positions to the exon-intron boundaries.

**Figure 4:**
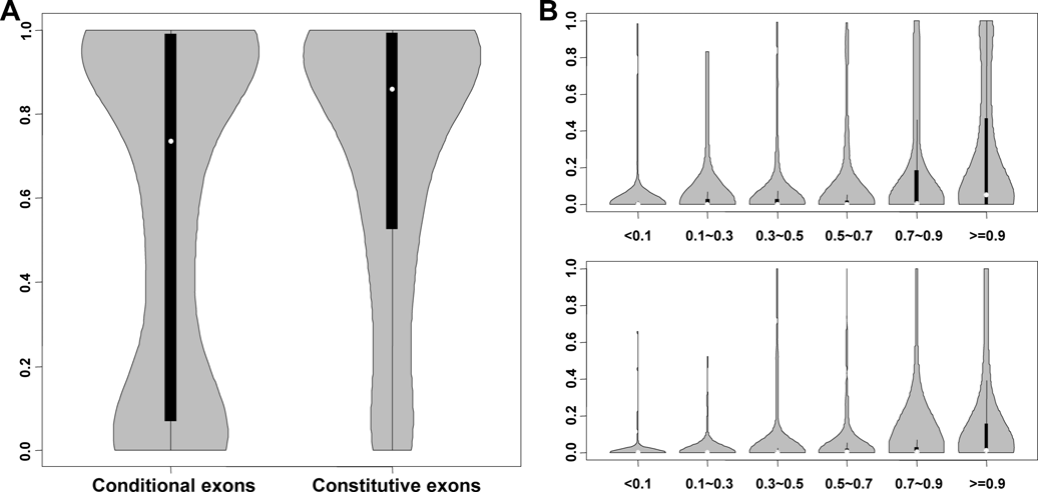
Violin plots of conservation scores of conditional exons and constitutive exons. (A) Exon region conservation. For each exon, the average conservation score across different positions was used (150 positions close to the 5′ edge and 150 positions close to the 3′ edge). (B) Relationship between exon region conservation and upstream intronic region conservation. Exons were divided into six groups according to their exonic conservation scores (on X axis). Y axis is the conservation score for the upstream intronic region. The upper panel is for conditional exons and the lower panel is for constitutive exons.

We further consider whether there are subpopulations among those conditional exons. We suspect that conditional exons can be divided into two groups: functional or non-functional exons. The assumption is that for those conditional exons with function, their conservation scores are relatively high and they tend to be highly regulated. The conservation scores of their regulatory upstream regions are also high. However, for those conditional exons produced by alternative splicing as random events, their conservation scores are low and the upstream intronic regions tend to be less conserved. Figure 4A shows the violin plots of the conservation scores of conditional exons and constitutive exons. Violin plot is similar to boxplot except that it adds the kernel density plot of the data. It clearly shows that the distribution of conservation scores of conditional exons is bimodal. Some of the conditional exons have high conservation scores and some of them have very low conservation scores. We next consider whether the upstream intronic regions of those highly conserved conditional exons are more conserved. Figure 4B shows the relationship between the conservation level of exon region (X axis) and the conservation level of upstream intronic region (−46, −18) (Y axis) for conditional exons (upper panel) and constitutive exons (lower panel). For both constitutive exons and conditional exons, if the conservation score of exon region is high, the upstream intronic region is more conserved. Compared with constitutive exons, conditional exons with the same conservation scores tend to have more conserved upstream intronic regions. It indicates that they may be highly regulated and most likely that they are functional.

### Discover Novel Conditional Exons from Intron Sequences

We used the Random Forests to learn the classifier for conditional exons and intron sequences. The Random Forests consist of many decision trees and each tree is constructed by a bootstrap sample from the original data. A decision tree can be treated as a set of Boolean functions of features and these conjunctions of features partition training samples into groups with homogenous class label. The output of the Random Forests for each test sample is the class with majority votes from these trees. The Random Forests generates an internal unbiased estimate of classification error based on the out-of-bag data during the Forests building process. There is no need for cross-validation or a separate test data. In this study, the high-quality training data were from the GENCODE project whose ultimate goal is to identify all protein-coding genes in the human ENCODE regions. We assembled 1,185 conditional exons and 4,490 intron sequence fragments with length from 30 to 330 bp. The 330 features we used were conservation scores of positions: 0, …, 164, −164, …, −0. The classification error rate is 0.10, the sensitivity is 0.64, the specificity is 0.97, and the false discovery rate is 0.15. We also considered the area under the curve (AUC) score that is the value of the area under the receiver operating characteristic (ROC) curve. AUC score is a global performance measure by combining both the sensitivity and the specificity. A perfect classifier will have AUC = 1 and a random classifier will have AUC = 0.5. Using the position-specific conservation score, we achieved an AUC score of 0.86.

We assembled 28,324,910 overlapped potential conditional exons (see Materials and Methods) from the intron regions of RefSeq gene collection. These potential exons are 30 to 330 bp long and flanked by AG and GT dinucleotides (the splice sites of introns). They have a poly-pyrimidine tract in their upstream regions. And they don't introduce in-frame stop codons. Those potential exons were tested using the trained classifier from the Random Forests. It resulted in 1,273,698 conditional exons. Because these predicted conditional exons may be overlapped, we focused on introns with at least one predicted conditional exon instead. About 21% (39,640/185,233) of tested introns contain at least one predicted conditional exon. However, for the ENCODE regions, about 16% of introns contain at least one conditional exon (excluding terminal exons). It indicates either a high false discovery rate in the discovered conditional exons or the incomplete annotation for the ENCODE regions. Indeed, It has bee reported that 59∼74% of human genes are alternative spliced and the cassette exons make up 53∼61% portion of alternative splicing events [1]–[4]. Our results that 21% of known introns contain at least one conditional exon narrow the gap between current annotation and experimental predication. By comparing the annotations of Refseq transcripts themselves, 4,774 introns are concluded to contain at least one conditional exon. Our Random Forests classifier predicted 76% of them (3,643/4,774). Based on a larger transcript annotation set (Refseq [15], UCSC [16] and Ensembl genes [17]), among the 185,233 tested RefSeq introns, 13,759 introns contain at least one conditional exon. And our Random Forests classifier predicted 58% (7,967/13,759) of them. The Random Forests classifier predicted another 31,673 introns containing conditional exons which can not be inferred from RefSeq, UCSC and Ensembl annotations.

**Figure 5:**
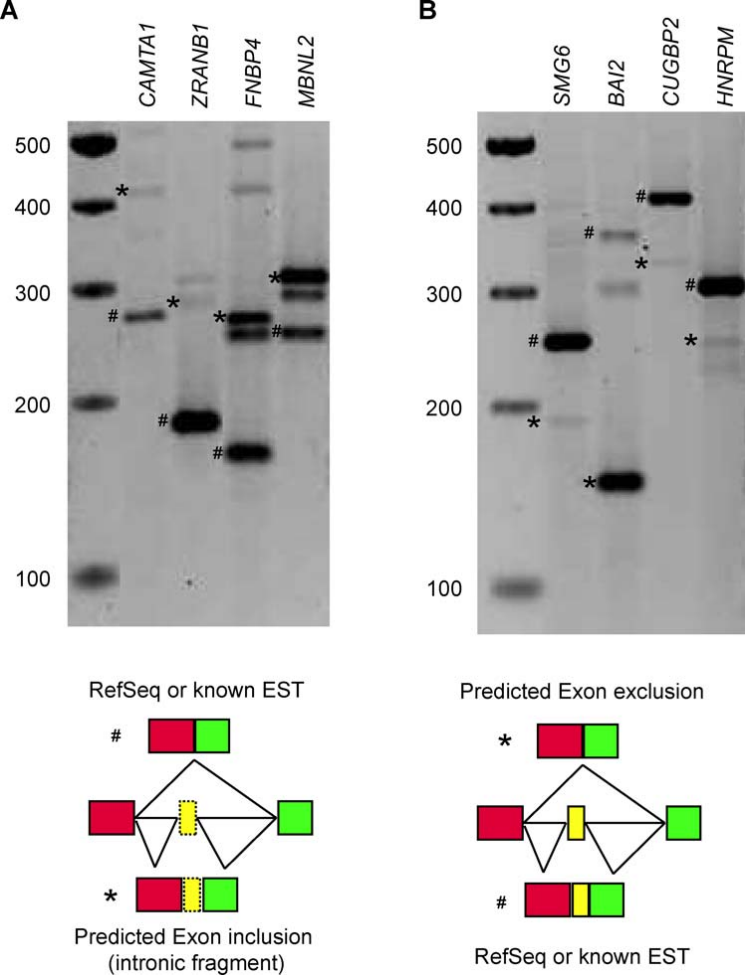
Experimental validation for some of predicted novel conditional exons. (A) RT-PCR shows that *CAMTA1*, *ZRANB1*, *FNBP4* and *MBNL2* contain conditional exons which were previously annotated as intron regions. The cell lines used were HeLa, HeLa, LA-N-5 and SHSY5Y cells respectively. PCR bands marked with “#” are transcripts of corresponding annotated RefSeq sequences. PCR bands marked with “*” are transcripts subject to sequencing and proved to contain or overlap with the predicted conditional exons from intron regions. The left lanes show the DNA molecular weight markers and their size in bp. (B) RT-PCR results show that *SMG6*, *BAI2*, *CUGBP2* and *HNRPM* express the predicted transcripts which exclude *de novo* identified conditional exons in LA-N-5, LA-N-5, SHSY5Y and LA-N-5 cells. PCR bands marked with “#” are transcripts of the corresponding annotated RefSeq sequences. PCR bands marked with “*” are transcripts that exclude the predicted conditional exons (proved by sequencing). The left lanes show the DNA molecular weight markers and their size in bp.

We used RT-PCR to test our predictions. We designed primers in the exonic regions flanking the introns which were predicted to contain conditional exons. These primers were screened against the provided library of human sequence repeats and have a high melting temperature (>60°C) to minimize non-specific amplification. Since many alternative splicing events occur in a tissue-specific manner, we harvested total RNA samples from five different human cell lines: LA-N-5, WERI, HeLa, HEK 293 and SHSY5Y cells. We randomly picked 15 introns from the top predictions (all of classification trees vote for conditional exons instead of introns) and designed specific primer pairs targeting exons which flank these introns. By RT-PCR, eleven primer pairs of fifteen yielded only one or no amplicons in these five cell lines. But the other four showed additional amplicons of higher molecular weight in one or more cell lines, indicating alternative transcripts with exon inclusion (Fig. 5A). Sequencing of these amplicons proved that they partially overlap with the predicted conditional exons.

### Features Distinguishing Conditional Exons and Constitutive Exons

**Figure 6:**
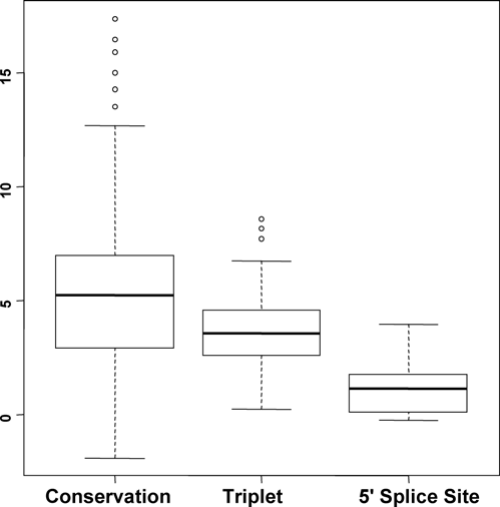
Boxplots of importance measures of conservation-score features, triplet-count features and 5′-splice-site features. Y axis is the importance measures that are the standardized importance score (z-score) from the Random Forests classifier. The “conservation” features include position-specific conservation scores of exons, upstream and downstream 100 bp intronic regions. The “triplet” features include 3-mer word frequencies for exons, upstream and downstream regions. The “5′ splice site” features include position-dependent single base counts at 5′ splice site for −3 to +6 positions (excluding +1 and +2 invariant positions). In addition, the importance measure is 7.4 for “exon length”, 0.3 for “length divisibility by 3”, and 2.4 for PPT intensity (not shown in the figure).

Next we consider how to classify known exons into conditional exons and constitutive exons. Besides conservation scores of exon positions 0, …, 149, −149, …, −0 and conservation scores of upstream and downstream 100 bp intronic regions, we also considered features used in [18]: (1) exon length; (2) exon divisibility by 3; (3) 3-mer word frequencies for exons, upstream 100 bp and downstream 100 bp intronic regions; (4) position-dependent single base counts at 5′ splice site (−3 to +6 positions, excluding +1 and +2 invariant positions); (5) intensity of the poly-pyrimidine tract (PPT) that is the number of pyrimidines in a 15 bp window of the last 19 nucleotides of the upstream intron (not including the last 4 nucleotides of the intron). The ENCODE training data show that the conservation scores of exon positions 0∼112, −119, −118, −102∼−0 are significantly different between conditional exons and constitutive exons with t test p-values≤0.01 (the median p-value is 6.2×10^−14^). The conservation scores of upstream intronic positions (−98∼−95, −93∼−87, −68∼−13, −3∼−1) and downstream intronic positions (1∼5, 9∼58, 60∼66, 69∼82, 84∼86, 94∼96) are significantly different between conditional exons and constitutive exons (the median p-value is 2.4×10^−4^). Table 1 lists other significant features with p-values≤0.01 by comparing conditional exons and constitutive exons. Those 3-mer words and the 5′ splice site positions may be related to splicing *cis* elements. The exon length and exon divisibility by 3 are not significant with a t test p-value 0.26 and a Fisher's exact test p-value 0.15. Figure 6 shows the boxplots of importance measures of features. The importance measure is estimated by the Random Forests. It is the raw importance score divided by its standard error (z-score). The raw importance score is determined by comparing the training data and the simulated data in which the considered feature is randomly permuted and other features are kept intact. The results indicate that the position-specific scores are the most important features.

**Table 1:** Features besides position-specific conservation scores that are significantly different between conditional exons and constitutive exons (p-values≤0.01).

Feature	P-value
eACC	3.38×10^−3^
eAGA	9.30×10^−3^
eTAG	1.97×10^−4^
eTCC	5.60×10^−5^
eTCG	4.64×10^−5^
eTGA	1.37×10^−4^
eCAA	3.73×10^−3^
eCTC	6.03×10^−4^
eCCT	6.06×10^−3^
eCGA	9.69×10^−3^
eGAA	8.18×10^−4^
eGAC	1.23×10^−5^
eGGG	6.15×10^−3^
in1TGA	7.02×10^−3^
in2CCA	5.72×10^−3^
T3	4.97×10^−3^
A4	5.18×10^−3^
C4	2.55×10^−3^

The first 15 p-values are based on t tests and the last three p-values are based on Fisher's exact tests. eACC, …, eGGG are the ACC, …, GGG frequencies in exon regions. in1TGA is the frequency of TGA in the upstream 100 bp intronic region. in2CCA is the frequency of CCA in the downstream 100 bp intronic region. T3, A4, C4 are position-dependent single base counts at 5′ splice site for position +3,+4 and +4.

Based on the training data from the ENCODE regions, although the specificity is high (0.95), the sensitivity is low (0.32), the AUC score is 0.73, the FDR is 0.27, and the classification error rate is 0.25. We applied this classifier to exons assembled from RefSeq genes (excluding 5′ terminal and 3′ terminal exons). A total of 8,813 out of 162,941 exons were labeled as conditional exons. Comparing the annotations of Refseq gene themselves, we can identify 4,255 conditional exons (excluding terminal exons). Our Random Forests classifier predicted 36% (1,524/4,255) of them. The sensitivity is close to our estimate from the training data (0.32). Considering the combination of Refseq, UCSC and Ensembl gene annotations, we can infer 20,930 conditional exons (excluding terminal exons). Our Random Forests classifier only predicted 17% (3,519/20,930) of them. The Random Forests classifier predicted another 5,294 conditional exons that cannot be inferred from gene annotations. The FDR for our training data is 0.27. Using the FDR and the sensitivity, the total number of conditional exons in the Refseq genes can be estimated as 8,813×(1.00−0.27)/0.32 = 20,105. Therefore, about 12% (20,105/162,941) exons are conditional exons. Given the large fraction of genes with alternative splicing (59∼74%) and the large fraction of cassette exon events (53∼61%), this estimate is reasonable.

We continued to test our prediction using RT-PCR and sequencing. We used exon array data across different tissues (available on the Affymetrix website http://www.affymetrix.com/) to roughly determine whether a gene was expressed in a specific tissue before we selected predicted transcripts for validation. Primers in the exon regions flanking predicted conditional exons were designed for RT-PCR experiments. These primers were also screened against the provided library of human sequence repeats and have a high melting temperature (>60°C) to minimize non-specific amplification. Of five exons we tested (the five exons are on the top prediction list), four have apparent PCR products excluding the predicted conditional exons (Fig. 5B), while the fifth does not yield any PCR product maybe due to low expression level. Sequencing of these alternative PCR transcripts proved that they are exactly the predicted conditional exons. In addition to the novel predicted conditional exons, examples of known conditional exons which were predicted by our methods were shown in Supplementary Figure S1.

### Ontology Study for Genes with Many Conditional Exons

After we predicted conditional exons, we were interested to know whether there is any functional characteristic of genes enriched with conditional exons. For each RefSeq gene, different transcripts were combined to assemble non-redundant introns and exons. For those introns, we counted the frequency of them containing conditional exons based on our predictions. For those exons, we counted the frequency of them being conditional exons based on our predictions. A total of 837 genes have ≥15 introns+exons and ≥20% of those introns and exons are related to conditional exons. David Functional Annotation tool (2008) [19] was applied to analyze gene annotations. Table 2 lists the significant gene annotation terms with at least 10 gene counts and the p-value after Bonferroni's correction≤0.001. Bonferroni's correction is a very stringent multiple comparison correction. Here it controls the probability of having one or more falsely declared significant annotation term≤0.001. The term “alternative splicing” is a UniProt knowledgebase keyword meaning “protein for which at least two isoforms exist due to distinct pre-mRNA splicing events”. It is the rank one significant gene annotation with Bonferroni corrected p-value 3.0×10^−62^. The UniProt sequence feature “splice variants” is also enriched with Bonferroni corrected p-value 1.6×10^−44^. Other significant annotations include GO terms related to nervous system development, synapse, protein binding, transcription factor activity, etc. This is consistent with the idea that development and signaling pathways are thought to involve a large number of alternative splicing events [20], [21].

**Table 2:** Enriched annotation terms for genes with many predicted conditional exons.

Category	Term	Count	Corrected P-value
SP_PIR_KEYWORDS	alternative splicing	438	3.0×10^−62^
UP_SEQ_FEATURE	splice variant	337	1.6×10^−44^
SP_PIR_KEYWORDS	chromosomal rearrangement	43	5.9×10^−13^
GOTERM_CC_ALL	synapse	42	9.6×10^−13^
SP_PIR_KEYWORDS	synapse	36	2.0×10^−12^
GOTERM_MF_ALL	binding	601	4.8×10^−12^
GOTERM_MF_ALL	protein binding	389	5.4×10^−12^
SP_PIR_KEYWORDS	phosphoprotein	281	1.5×10^−11^
SP_PIR_KEYWORDS	activator	55	6.4×10^−11^
GOTERM_CC_ALL	synapse part	29	1.8×10^−10^
GOTERM_BP_ALL	developmental process	213	4.3×10^−10^
SP_PIR_KEYWORDS	cell junction	43	2.6×10^−9^
GOTERM_BP_ALL	biological regulation	298	1.2×10^−8^
GOTERM_BP_ALL	multicellular organismal development	163	1.3×10^−8^
GOTERM_BP_ALL	anatomical structure development	152	2.6×10^−8^
GOTERM_BP_ALL	regulation of biological process	275	3.5×10^−8^
GOTERM_CC_ALL	cell junction	48	5.3×10^−8^
GOTERM_MF_ALL	transcription regulator activity	115	1.0×10^−7^
GOTERM_BP_ALL	nervous system development	74	1.2×10^−7^
INTERPRO	Extracellular ligand-binding receptor	15	1.7×10^−7^
SP_PIR_KEYWORDS	Transcription regulation	125	1.8×10^−7^
GOTERM_CC_ALL	postsynaptic membrane	24	2.1×10^−7^
SP_PIR_KEYWORDS	Transcription	126	2.5×10^−7^
SP_PIR_KEYWORDS	Postsynaptic cell membrane	22	3.1×10^−7^
GOTERM_MF_ALL	glutamate receptor activity	16	4.1×10^−7^
GOTERM_MF_ALL	ionotropic glutamate receptor activity	11	9.0×10^−7^
GOTERM_BP_ALL	system development	126	1.4×10^−6^
INTERPRO	NMDA receptor	11	1.5×10^−6^
INTERPRO	Glutamate receptor-related	11	1.5×10^−6^
INTERPRO	Ionotropic glutamate receptor	11	1.5×10^−6^
KEGG_PATHWAY	Axon guidance	26	1.6×10^−6^
SMART	PBPe	11	1.8×10^−6^
GOTERM_MF_ALL	extracellular-glutamate-gated ion channel activity	11	3.5×10^−6^
GOTERM_BP_ALL	multicellular organismal process	220	8.5×10^−6^
GOTERM_BP_ALL	cell differentiation	126	1.9×10^−5^
GOTERM_BP_ALL	cellular developmental process	126	1.9×10^−5^
GOTERM_BP_ALL	regulation of cellular process	248	2.3×10^−5^
GOTERM_MF_ALL	transcription factor activity	80	5.0×10^−5^
SP_PIR_KEYWORDS	dna-binding	116	5.5×10^−5^
SP_PIR_KEYWORDS	repressor	36	6.3×10^−5^
GOTERM_MF_ALL	extracellular ligand-gated ion channel activity	17	9.5×10^−5^
GOTERM_CC_ALL	neuron projection	19	1.9×10^−4^
GOTERM_BP_ALL	regulation of metabolic process	177	2.8×10^−4^
GOTERM_BP_ALL	positive regulation of transcription	37	3.2×10^−4^
GOTERM_BP_ALL	cell development	91	3.2×10^−4^
GOTERM_BP_ALL	regulation of nucleobase, nucleoside, nucleotide and nucleic acid metabolic process	161	4.7×10^−4^
GOTERM_BP_ALL	regulation of transcription	158	5.2×10^−4^
GOTERM_BP_ALL	synaptic transmission	35	5.5×10^−4^
GOTERM_BP_ALL	cell communication	228	6.0×10^−4^
GOTERM_BP_ALL	positive regulation of nucleobase, nucleoside, nucleotide and nucleic acid metabolic process	37	6.8×10^−4^
GOTERM_BP_ALL	regulation of gene expression	165	7.3×10^−4^
GOTERM_MF_ALL	transcription activator activity	35	8.6×10^−4^
GOTERM_BP_ALL	regulation of cellular metabolic process	170	8.6×10^−4^
GOTERM_BP_ALL	regulation of transcription, DNA-dependent	149	9.0×10^−4^

Those gene annotation terms have at least 10 gene counts and p-values after Bonferroni's correction≤0.001. The p-value is from David Funtional Annotation tool (2008) and it is based on a modified Fisher's exact test. The annotation terms considered are from the default settings.

## Discussion

In this study, we characterize important features of position-specific conservation scores across conditional exons, constitutive exons and introns through the thoroughly annotated ENCODE genomic regions. Based on such important distinct features, we predicted many novel conditional exons which were previously known to be constitutive exons and predicted many introns which contain conditional exons. Some of these predictions were validated by RT-PCR followed by sequencing. Our comparative genomics approach is an important complement to current experimental technologies in identifying alternative splicing events at the genomic scale. In addition, our novel predictions provide an immediate interest of adding corresponding probes into exon arrays and exon-exon junction arrays.

In this paper, we found that constitutive exons have higher conservation level in exon regions and lower conservation level in flanking intron regions compared to conditional exons. This is based on the high-quality annotations of ENCODE regions and the conservation scores estimated from the alignment of 17 vertebrates. Some groups reported opposite results [7], [22]. Looking at each literature's methods carefully, we found that such discrepancy was mainly due to different sampling of training data. When sampling alternative exons, Sorek's and Yeo's papers [7], [22] selected human-mouse orthologous exons both of which are flanked by splice sites. They further required that alternative splicing events occur in both human and mouse. Such sampling had undoubtedly achieved very high conservation level of “alternative exons”. These “alternative exons” maintain conserved sequences for human-mouse orthology and conserved regulatory sequence elements for conserved alternative splicing. In contrast, our sampling of conditional exons and constitutive exons did not take into account of neither human-mouse orthology nor conserved splicing events between human and mouse. Our sampling completely relied on the high-quality ENCODE annotation data instead. This allows the hypothesis that human genome and mouse genome evolve independently to create different alternative splicing events. And it has no bias of assembling “conserved sequence” to study conservation level. We also found that the distribution of conservation scores of conditional exons is bimodal. Some of the conditional exons have high conservation scores and some of them have very low conservation scores (Fig. 4A).

Most importantly, we found that the differences of conservation scores are position-dependent. The position-specific conservation scores of exons and their flanking intronic regions may reflect functional splicing *cis* elements. The differences in position-specific conservation between conditional exons and constitutive exons and their flanking intronic regions may provide us information about the subtly different, if not significantly different splicing mechanisms for conditional exons and constitutive exons. For the exon region, the differences between conditional exons and constitutive exons are remarkably more significant in regions near the boundaries (Fig. 2). This could indicate that constitutive exons have stronger splicing signals at the boundary and for instance facilitate exon definition during splicing. For their flanking intronic regions, the upstream intronic regions and the downstream intronic regions are not symmetric. The conservation differences for upstream intronic regions are larger than those for downstream intronic regions. These may indicates that upstream intronic regions are more important than downstream intronic regions in regulating functional alternative splicing. Particularly, the upstream −46 to −18 bp intronic regions of conditional exons are significantly more conserved than those of constitutive exons (Fig. 3). The enriched sequence motifs in these regions may participate in the alternative splicing modulation. In addition, we classified sub-populations of conditional exons. Some conditional exons are conserved and have highly conserved upstream intronic regions (Fig. 4), which indicate that they may be highly regulated and functional. Some conditional exons are less conserved and lack the highly conserved upstream regions (Fig. 4). They may just be the products of random splicing events or newly evolved splicing event. It is also noteworthy that in all introns, the (−47, −7) region close to 3′ splice site are more conserved than its corresponding (7, 47) region close to the 5′ splice site. Such asymmetry seems consistent with the importance of polypyrimidine tract right upstream of the 3′ splice site.

Based on the high-quality training data set, the Random Forests classifier achieved specificity as high as of 0.97 and a sensitivity of 0.64 for conditional exon prediction from intron regions. For the classification of conditional exons from the current exon list, if we only use the position-specific conservation score, the classification error rate is about 25.2%. If we ignore the position-specific effect and use the average conservation score of exon regions, upstream and downstream intronic regions, the error rate increases to 30.3%. Adding other features such as triplet counts and others improves the classifier a little (error rate decreases from 25.2% to 25.0%).

Compared with Sorek et al.'s studies [22], in our training data the exon length and exon divisibility by 3 are not significant. Although the exon lengths of conditional exons are less than those of the constitutive exons (average 170.4 bp vs. average 182.2 bp), the difference is not statistically significant with a t test p-value 0.26 (one-tailed p-value 0.13). Although the portion of exons whose length is a multiple of 3 among conditional exons is slightly larger than that among constitutive exons (42% vs. 40%), the Fisher's exact test p-value is 0.15 (one-tailed p-value 0.08). There are several possible reasons: first, the scope of alternative exons and constitutive exons that we studied is different. We focus on conditional exons and constitutive exons. The conditional exons include cassette exons, mutually exclusive exons, retained introns and other complicated alternative splicing events. Sorek et al's cassette exons were those exons included and skipped in one or more transcripts, and the boundaries of both 5′ and 3′ flanking exons are shared in the transcripts that include and skip that exon, and the skipping events happen both in human and mouse. Their definition of constitutive exons was those that are supported by at least four expressed sequences, with no skipping event, both in human and mouse. These dramatically narrowed down the scope of either alternative exons or constitutive exons. It has been reported that there are slightly more exons whose length are exact multiple of three for alternatively spliced exons. However, orthologous exons that are alternatively spliced in multiple organisms showed a substantially increased bias to be exact multiple of three in length [23], [24]. Our definition of conditional exons does not require them to be orthologous exons so that the selection pressure for protein reading frame preservation is relatively low. Secondly, the selection of training data is different. We used thoroughly annotated ENCODE regions. Sorek et al used 243 alternative and 1,753 constitutive exons that are conserved between human and mouse. After all, the 1,753 constitutive exons may still contain a handful of alternative exons which have not been discovered by meticulous experiments. In the process of validating our predicted conditional exons, we found that in most cases the novel predicted transcripts are either not expressed or expressed at a much lower level than those of known transcripts. This might be one of the reasons why they have not been discovered by previous EST sequencing. It may also be the reason for the low validation rate (4 out of 15) in the case of the prediction from intron regions. In a world of alternative splicing, it is difficult to disprove an alternative splicing possibility. This problem may still exist in the ENCODE annotation, but to a lesser degree presumably.

Our method has much broader scope and application than previous alternative exon prediction algorithms. For example, Yeo et al. focused on alternative splicing events conserved in human and mouse [7]. Their training sets were limited to orthologous human-mouse exon pairs with conserved splicing patterns. Secondly, only orthologous human-mouse exons (∼100k) are eligible for their prediction program. Thirdly, their approach can not predict novel exon inclusion events, or splicing events from regions currently annotated as introns. Our prediction of exon skipping events does not rely on the occurrence of its orthologous exon skipping in another organism. In addition, we can predict novel exons from intron regions.

Finally, although we achieved a high specificity (97% and 95% for the identification of novel exons from introns and the identification of conditional exons from current exon list respectively), the sensitivity is still not satisfying (64% for identifying novel conditional exons from intron sequences, 32% for identifying conditional exons from current exon list). Future work will need to explore more features which can differentiate conditional exons, constitutive exons and introns.

## Materials and Methods

### Position-specific Conservation

The high-quality manual annotations in the ENCODE regions were generated by the GENCODE project and were downloaded from the UCSC Genome Browser (http://genome.ucsc.edu/). Non-redundant middle exons (excluding 5′-terminal and 3′-terminal exons) and introns were used for further analysis. A total of 4,187 exons and 5,749 introns were assembled. If one exon is located in the intron region of other transcripts, it is called a conditional exon. Otherwise, it is called a constitutive exon. A total of 1,314 conditional exons and 2,873 constitutive exons were identified. If an intron doesn't contain a conditional exon, it is called a conditional-exon-free intron. A total of 4,800 such introns were identified. We note that constitutive exons and conditional-exon-free introns may still have other types of alternative splicing such as alternative 3′ or 5′ splice sites.

The conservation score based on a phylogenetic hidden Markov model for 17 vertebrates [11] was downloaded from the UCSC Genome Browser. The score of each site is the posterior probability that the site is in the conserved state of the phylogenetic hidden Markov model. For every site of exon, define x as the position relative to the nearest splice site. It is positive for distances from the 5′ exon edge and negative for distances from the 3′ exon edge. For example, an exon with length 50 contains positions 0, … , 24, −24, …, −0. Similarly, x can be defined for introns. The conservation score can be found for each x as s(x).

### Training Data

The training data for identifying novel conditional exons from introns were prepared as following. A total of 1,185 conditional exons with length from 30 to 330 bp in the ENCODE regions were used as training data. The lengths were recorded as (L_1_, L_2_, …, L_1,185_). For each conditional-exon-free intron with length≥100 bp in the ENCODE regions, we randomly picked up a fragment with length sampled from (L_1_, L_2_, …, L_1185_). Therefore, those sampled intron fragments also have length from 30 to 330 bp. In total, 4,490 intron fragments were created and used as training data. In addition, a total of 1,314 conditional exons and 2,873 constitutive exons in the ENCODE regions were used as the training data for classifying exons into conditional exons and constitutive exons. The training exons and introns can be found in Supplementary Table S1, S2, S3.

### Random Forests Learning

Random Forests machine learning [14] was used to learn the classifier. The code was downloaded from (http://www.stat.berkeley.edu/breiman/RandomForests/cc_home.htm). We built 1,000 trees for each Random Forests. At each node, the number of variables we considered is the square root of the total number of features. The features for identifying novel conditional exons from introns are the position-specific conservation scores: s(0), …, s(164), s(−164), …, s(−0). Thus, we have 330 features. Denote true positive number as TP, false negative number as FN, true negative number as TN, and false positive number as FP. The classification error rate: (FN+FP)/(TP+FN+TN+FP), sensitivity: TP/(TP+FN), specificity: TN/(TN+FP), and false discovery rate (FDR): FP/(TP+FP) were recorded. The area under the curve (AUC) score is calculated as
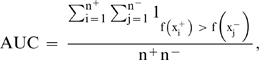
Where n^+^ is the number of positive samples (e.g., conditional exons), n^−^ is the number of negative samples (e.g., constitutive exons), x^+^ are the features for positive samples, x^−^ are the features for negative samples, f(·) is the scoring function (e.g. the number of votes for conditional exons), and 1_(·)_ is the indicator function.

The features for classifying exons into conditional exons and constitutive exons include: position-specific conservation scores of exon positions (0, …, 149, −149, …, −0), upstream 100 bp regions (−100, …, −1) and downstream 100 bp regions (1, …, 100); exon length; exon divisibility by 3 (1: yes, 0: no); 3-mer words frequencies for exon, upstream 100 bp region and downstream 100 bp region; position-dependent single base counts at 5′ splice site for −3 to +6 positions (excluding +1 and +2 invariant positions); intensity of the poly-pyrimidine tract (PPT) which is the number of pyrimidines in a 15 bp window of the last 19 nucleotides of the upstream intron (not including the last 4 nucleotides of the intron). Those non-conservation score features were also used in Dror et al.'s paper [18]. In their paper, instead of using position-specific conservation scores, they used the percent identity when aligned to the mouse counterpart.

### Test Data

Known protein-coding genes from the NCBI mRNA reference sequences collection (RefSeq) [15] were downloaded from the UCSC Genome Browser (Build hg18). Introns were assembled from these genes. If two introns share the same positions but they have different phases, they were still treated as two introns. In other places of the paper, if two introns share the same positions, we treated them as redundant introns. For each intron, we scanned it from 5′ to 3′ to identify possible exons: (1) with length from 30 to 330 bp; (2) they are flanked by AG and GT dinucleotides; (3) the intensity of PPT is ≥9; (4) they will not cause in-frame stop codons. Those procedures resulted in 28,324,910 fragments. Conservation scores were assigned to every position of those fragments. The fragments were classified as conditional exons or introns according to the classifier we learned from the training data (with ≥50% trees voting for conditional exons or introns). For the classification of conditional exons from current exon list, we assembled 162,941 unique exons from RefSeq (excluding terminal exons). They were classified into conditional exons and constitutive exons according to the classifier learned from the training data. The test exons and introns can be downloaded from http://www-rcf.usc.edu/liangche/research/rfexon/.

### Tissue Cell Culture and RNA Preparation

LA-N-5, HeLa, SHSY5Y, WERI and HEK 293 cell lines were cultured following standard guidelines provided by American Type Culture Collection. Total RNA samples of these cell lines were prepared using Trizol according to manufacturer's protocol (Invitrogen, CA).

### Primer Design and RT-PCR

Primer design was done with the Primer3 online software (http://frodo.wi.mit.edu). Sequence assembly of a tested transcript includes the predicted conditional exon and its flanking exons and the product should include the predicted conditional exon. Primers are filtered against mispriming human libraries and have a high melting temperature (>60°C) to minimize non-specific amplification. Primer sequences are as followed.

*CAMTA1*: AGAGGCACCGCTGGAACACT (forward), TGGGGATGATGGAGGAATGG (reverse); *ZRANB1*: GCTGTGGGAAGCAAGGAGGA (forward), ATCTGCGGTGAGCTGACGTG (reverse); *FNBP4*: CGGGAAGGGGCTCTTAATGG (forward), GACTCGCCCGACTGTTCGTT (reverse); *MBNL2*: AGAGACCGACTGCCGCTTTG (forward), TGAAGAGCACCAGGGGGAAA (reverse); *SMG6*: CCATCCCATCCACGGTCTTC (forward), TAAGCTGCAGCATGCGGGTA (reverse); *BAI2*: GGTCCCCGACTTAGGGATGG (forward), AGGCGCAGGGACAGAATCAC (reverse); *CUGBP2*: GGACCTGATGGGCTGAGTCG (forward), CATTGGTGCTGGTGGCTGAG (reverse); *HNRPM*: AGGAGGCAATCGCTTTGAGC (forward), GCATTGCTCTCCTGGCATGTT (reverse).

The RT reaction was done following manufacturer's instruction (Invitrogen, CA). 1 µg of total RNA and 50 ng of random hexamer were used for one RT reaction. After RT, 3 µl of first-strand cDNA were used for one PCR reaction (50 µl). A program of 30 cycles of melting (30s at 94°C), annealing (30s at 60°C), and extension (1 min at 72°C) was used.

### PCR Product Extraction and Sequencing

PCR products were separated by electrophoresis on a 2% agarose gel supplemented with ethidium bromide and were visualized under a UV light. PCR products were extracted using Qiagen Gel Extraction kit (Qiagen, CA), ligated into pCR-TOPO vector and then transformed into chemically competent cells using TOPO TA Cloning Kit (Invitrogen, CA) according to manufacturers' instructions. Bacteria were plated on LB/x-gal/Amp agar plates and grown overnight at 37°C. A maximum of 3 colonies were picked from each plate, amplified and used for sequencing reaction (www.laragen.com) with forward M13 primers.

## Supporting Information

Figure S1 Examples of well known alternatively spliced genes. For gene *APP*, there are three RefSeq transcript isoforms: NM_000484, NM_201413 and NM_201414. Two exons (exon 7 and exon 8) are known to be included in some transcripts and spliced out for others. Our methods predicted both of them correctly. There are three RefSeq transcript isoforms for gene *GRIA2*: NM_001083619, NM_000826, NM_001083620. Two exons (exon 14, exon15) are known conditional exons. Our methods predicted both of them correctly. In addition, our methods predicted that exon 16 is a conditional exon.
(0.86 MB TIF)

Table S1 Training conditional exons assembled from the ENCODE annotations. They are middle exons (excluding 5′-terminal and 3′-terminal exons) and they are located in the intron region of other transcripts. All of them (1,314) were used to train the classifier to distinguish conditional exons and constitutive exons. A total of 1,185 conditional exons with length between 30 and 330 bp were used to train the classifier to distinguish conditional exons and intron sequences.
(0.01 MB ZIP)

Table S2 Training constitutive exons assembled from ENCODE annotations.
(0.02 MB ZIP)

Table S3 Training intron fragments with length from 30 to 330 bp. They were sampled from conditional-exon-free introns.
(0.03 MB ZIP)
