# Supplementary figures and images for: Identify Alternative Splicing Events Based on Position-Specific Evolutionary Conservation
Source: PLoS One. 2008 Jul 30;3(7):e2806. doi: 10.1371/journal.pone.0002806 (PMC2467489; doi:10.1371/journal.pone.0002806)

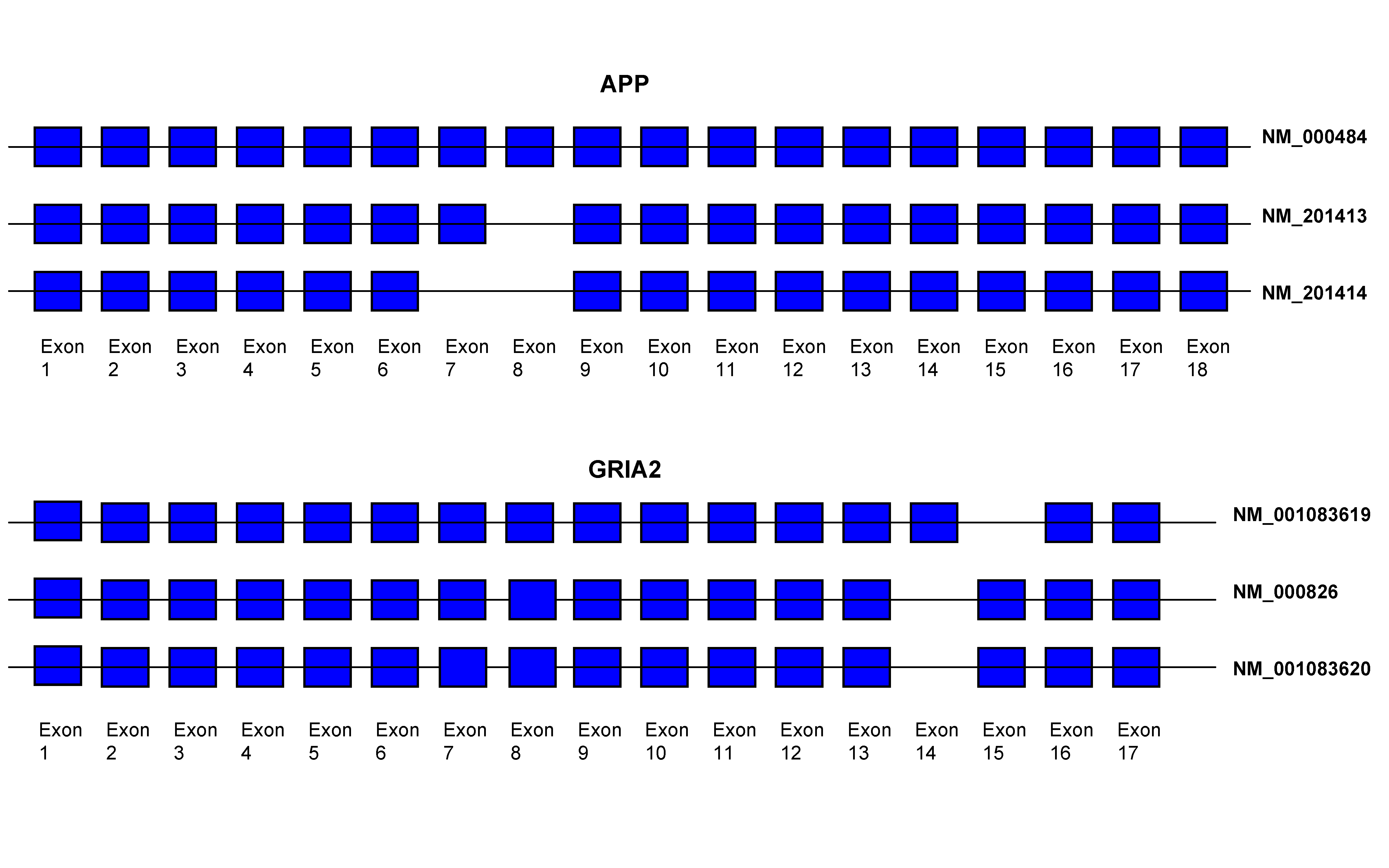

Supplement: Figure S1 — Examples of well known alternatively spliced genes. For gene APP, there are three RefSeq transcript isoforms: NM_000484, NM_201413 and NM_201414. Two exons (exon 7 and exon 8) are known to be included in some transcripts and spliced out for others. Our methods predicted both of them correctly. There are three RefSeq transcript isoforms for gene GRIA2: NM_001083619, NM_000826, NM_001083620. Two exons (exon 14, exon15) are known conditional exons. Our methods predicted both of them correctly. In addition, our methods predicted that exon 16 is a conditional exon. (0.86 MB TIF) [file pone.0002806.s001.tif]
